# Laser Treatment in Hirsutism: An Update

**DOI:** 10.5826/dpc.1002a48

**Published:** 2020-04-20

**Authors:** Yasmeen Jabeen Bhat, Safia Bashir, Nahida Nabi, Iffat Hassan

**Affiliations:** 1 Department of Dermatology, STD & Leprosy, Government Medical College, Srinagar, India

**Keywords:** hirsutism, laser, photothermolysis, epilation

## Abstract

Unwanted hair growth, which is a common aesthetic problem, has traditionally been treated using various techniques such as shaving, waxing, and epilation, but most of these provide only a temporary solution. Laser and light-based technology for hair removal has become one of the fastest growing procedures in modern cosmetic dermatology in the last decade. Clinical experience suggests that in the ideal subject with fair skin and dark hair, laser treatment can reduce hair growth significantly. This article reviews the various laser and light-based devices used for hair removal along with the various laser and patient parameters that affect the outcome of laser treatment for hair removal. Photoepilation, when properly used, offers clear advantages when compared with older, traditional techniques.

## Introduction

A laser is a device that controls the way in which energized atoms release photons. “Laser” is an acronym for “light amplification by stimulated emission of radiation,” which in itself describes the working of a laser. Lasers do not provide permanent hair removal, but they are popular because of selective hair damage, less time consumption, longer hair-free interval, and fewer side effects. A single treatment can reduce hair by 10%–40% and repeated treatments by as much as 90%, and these results may persist for as long as 12 months [1,2]. Laser hair removal is said to be permanent when there is a stable decrease in the number of terminal hair for a period longer than the complete hair growth cycle at a given site after treatment [2]. There are various factors related to technology and patient which could result in variable, unpredictable, or poor responses to laser hair removal in spite of ensuring appropriate indications and adequate parameters of laser use.

## Laser Physics Parameters

Laser hair removal is a multifactorial process that involves complex photothermal reaction via the epidermis to the dermal matrix, aimed to cause hair follicle damage while sparing the epidermis. Various laser parameters such as power, spot size, irradiation time, and repetition rate determine the final outcome in laser hair removal. Tissue parameters such as absorption and scattering coefficients, density, heat capacity, and thermal conductivity are equally important.

### Fluence

A proper fluence is of paramount importance in getting the required therapeutic effect in laser hair removal. While higher fluences can increase efficacy, they can also increase adverse effects. The right fluence is determined by the highest tolerable energy or a test patch that generates perifollicular erythema and edema [3].

With a diode laser, fluences in the range of 30–35 J/cm^2^ are adequate for type II-III skin. Lower fluences in the range of 20–24 J/cm^2^ are used for darker skin types [4]. Using suboptimal fluence is one of the most important causes of a poor response to laser hair removal.

With lower fluences, temporary rather than permanent removal is achieved. Roosen et al studied the effect of low-fluence photoepilation on hair follicles. Their findings suggest that transition of anagen follicles to catagen phase happens with low fluences [5]. Long-term effects of hair removal depend on a number of factors such as hair color, skin color, and the fluence tolerated by the patient.

### Spot Size

Spot size refers to the size of the laser probe or head that is used during a laser procedure. This in effect is the area over which the laser beam is delivered in a single shot of laser treatment. The importance of spot size lies in scattering of laser energy by collagen fibers outside the treatment zone. More photons are likely to be scattered if smaller spot sizes are used, while with larger spot sizes a higher percentage of photons are delivered to the skin and are likely to remain within the treatment area. One study showed a superior response to 18-mm spot size in comparison with 12-mm spot size in axillary hair removal [6].

### Pulse Duration

Optimal pulse duration in hair removal is calculated based on thermal relaxation time. For terminal hairs, the calculated thermal relaxation time is in the range of 100 milliseconds. The pulse duration thus used has to be in this range only. A subnormal therapeutic effect is usually caused by an inadequate pulse duration.

## Principle of Laser Hair Removal

The technology used in hair removal by lasers is based on the principle of selective photothermolysis. According to this principle, selective thermal destruction of a target will occur if sufficient energy is delivered at a wavelength well absorbed by the target within a time period less than or equal to the thermal relaxation time of the target. The thermal relaxation time is the time it takes for the target to cool (half of its baseline temperature) and transfer the heat to surrounding structures [7]. Under these conditions, it is possible to selectively target structures (eg, hair follicle) while sparing the surrounding structures or tissues. The target site for the selective destruction of hair follicles can either be endogenous melanin or exogenous chromophore. “Thermal damage time” rather than the thermal relaxation time has also been introduced as a new concept in laser hair removal. Ideal pulse duration for medium to coarse hair removal may be longer than the thermal relaxation time of the hair follicle as indicated by various studies. Dark hair that contains large amounts of eumelanin readily absorbs these wavelengths and is most susceptible to laser-induced damage [8].

## Factors Affecting Outcomes of the Laser Therapy

### Patient Factors

An ideal patient for conventional laser hair removal is one who has thick, dark terminal hairs, light skin, and normal hormonal status. Patient selection should not be compromised during laser hair removal as this can decrease response to treatment. The following sections explain these factors in detail.

### Skin Type

Light skin (Fitzpatrick skin type I-IV) and dark hair are an ideal combination for effective hair removal. Since the chances of epidermal melanin absorption are less with higher fluence, short pulse duration can be used [9,10]. The absorption is more at the level of follicular melanin rather than epidermal melanin, thus reducing the chances of epidermal damage. The long-pulsed Nd:YAG laser remains the recommended choice in very dark individuals and tanned patients because of its longer wavelength [11]. The safety of patients with type V-VI skin is a challenge for laser hair removal because of the high density of competing chromophore in the epidermis. A wavelength that is less absorbed by melanin may be less effective clinically as the target chromophore for hair removal laser is melanin in the hair bulb and bulge.

### Type of Hair, Color of Hair, and Stage of Hair Cycle

The anagen hair is more prone to laser therapy since melanin is present only in anagen hair. At any given time, almost 10%–15% of hair may be catagen or telogen stage; hence treatment has to be extended for many months to cover as many hair follicles in its anagen stage. Terminal hair, being more pigmented, responds better than vellus hair. Good response to laser hair removal occurs when the targeted hair has a high concentration of chromophores. Thin, fine hairs have less pigment and hence are poor choices for laser hair removal, even with best fluences and multiple treatments, compared with thick terminal hairs [12,13]. This is true when treating areas such as the upper lip, where chromophore in vellus hairs is less for laser wavelength absorption.

### Hormonal Profile

Patients should be evaluated for hirsutism by doing various hormonal assays, especially for testosterone levels, as these may influence the response to laser therapy. Polycystic ovarian syndrome, thyroid dysfunctions, adrenal hyperplasias, and hyperprolactinemia are hormonal dysfunctions that influence hair regrowth following laser hair removal [14,15].

During pregnancy there is an increase in the levels of prolactin. The hyperprolactin state has a melanocyte-stimulating effect. All light therapies are ineffective for hair reduction in cases of hyperprolactinemia due to pregnancy or amenorrhea galactorrhea syndrome, which upregulate melanocyte-stimulating hormone in stem cells of the hair. Hyperprolactinemic states are often associated with polycystic ovarian syndrome. Hirsutism that persists despite 6 or more months of monotherapy with an oral contraceptive demands additional pharmacological therapies. Adding an antiandrogen is justified for women who choose hair removal therapy by laser/photoepilation, desiring a more rapid initial response. Eflornithine cream can be added during treatment for women with known hyperandrogenemia who choose laser hair removal.

A trial of at least 6 months before making changes in dose, changing medication, or adding pharmacological therapy to minimize hair regrowth with lasers is justified. Concomitant hormone therapy in cases of cutaneous hyperandrogenism may overcome poor outcomes of a stand-alone laser hair removal procedure.

It is important to establish the etiology and treat the underlying cause of hirsutism. Peripheral androgen blockage can be achieved by suppression of underlying hormonal imbalance. This can further be combined with laser hair removal to optimize results. Oral contraceptives cause suppression of production of luteinizing hormone and follicle-stimulating hormone, leading to a decrease in ovarian androgen production and decrease in adrenal androgen. Low-androgenic progestins are preferred as they cause antagonism of 5α-reductase and androgen receptor. Insulin sensitizers such as metformin significantly decrease insulin resistance. Untreated hormonal diseases can result in variable-to-poor responses to laser hair removal, and these patients usually require more sessions than patients with normal hormone levels [16,17].

### Site of Hair Removal

**Figure 1:**
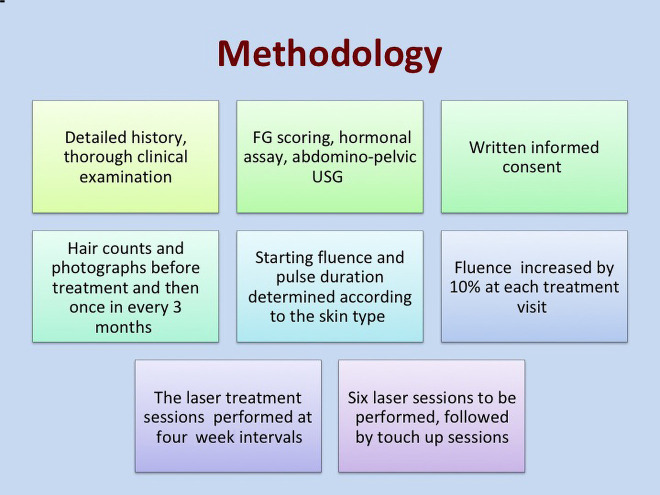
The procedure for laser hair reduction. FG = Ferriman-Gallwey; USG = ultrasonography.

Since there are differences in anagen-telogen ratios in various anatomic sites, there may be differences in response rates. Axillae and belt areas respond better than legs, arms, and chest. The procedure for laser hair removal is summarized in Figure 1.

## Grading of Efficacy

Efficacy is graded according to a 4-point visual scale ranging from poor to excellent depending on the percentage of hair reduction from baseline: poor: <25%; fair: 25%–50%; good: 50%–75%; excellent: >75%.

**Figure 2:**
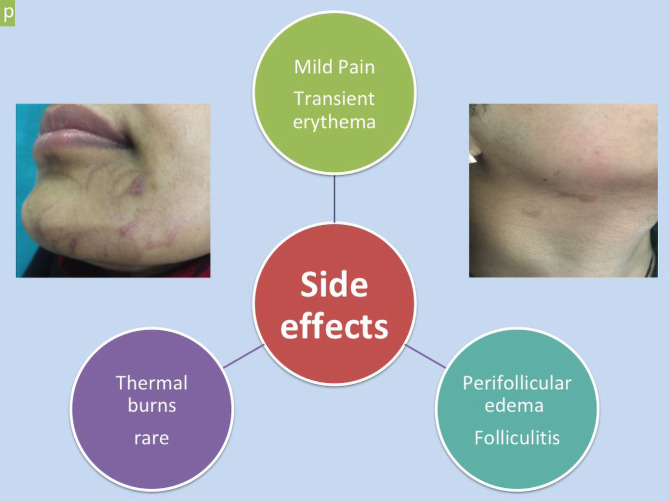
Side effects of laser hair removal.

Figure 2 shows the side effects of laser hair removal.

## Lasers Used in Hair Removal

More than 15 laser systems have been approved by the US Food and Drug Administration for use in hair removal. These systems include ruby (694 nm), alexandrite (755 nm), diode (800–1,000 nm), Q-switched and long-pulsed neodymium: yttrium-aluminum-garnet (Nd:YAG; 1,064 nm), and intense pulsed light sources (550–1,200 nm). A summary of commercially available lasers is given below.

### Normal-Mode Ruby Laser (694 nm)

The pulsed ruby laser was originally used to perform melanin-based selective photothermolysis of hair. The ruby laser delivers red light at a wavelength of 694 nm. Ruby lasers are particularly useful for light-skinned (Fitzpatrick skin types I-III) individuals with dark hair because of high melanin absorption at 694 nm. Allison et al studied the long-term hair regrowth in 3 patient groups: top lip (n = 25), axillae (n = 25), and legs (n = 9). Two treatments were given on the right and left sides at monthly intervals followed by a third treatment given randomly to one side. Hair counts of the experimental sites made at monthly intervals for 1 year indicated long-term hair reduction in all patients. A single treatment was seen to reduce hair counts by up to 75%. While 3 treatments had an impact for 2 additional months, no long-term effect was noted. Spontaneous hair reduction was unexpectedly found at 5 months after treatment and lasted 2 months [18]. Thus ruby laser produced a persistent two-thirds reduction in hair count over 8 months of follow-up and no significant regrowth follow-up to 12 months. Studies with short-term follow-up have observed 37%–72% reduction at 3 months after 1 to 3 treatments, to a 38%–49% hair reduction 1 year after 3 treatment sessions.

### Alexandrite Laser (755 nm)

The alexandrite laser allows greater depth of penetration, making it relatively safe in darker-skinned (Fitzpatrick skin types I-IV) individuals. However, melanin absorption is somewhat less at the wavelength of alexandrite (755 nm) when compared with the ruby (694 nm). The reported success rate of hair removal using the alexandrite has ranged from 40% to 80% at 6 months after several treatments [19].

### Diode Laser (800–1,000 nm)

Diode lasers for hair removal were cleared to market in the USA in 1997. Individuals with darker skin can be treated more safely with this system because of the longer wavelength, the active cooling, and the longer pulse widths. The diode laser system has been found to be better tolerated by patients with darker skin types (V-VI) than the ruby laser. Variable success rates ranging from 65% to 75% hair reduction at 3 months after 1 to 2 treatments with fluences of 10–40 J/cm^2^, to 75% hair reduction in 91% of individuals 8 months after 3 to 4 treatments at 40 J/cm^2^ have been reported with the diode laser system. Sadick et al studied 24 female subjects (skin types II-IV) treated 3 times at monthly intervals with a new 810-nm diode laser (spot size 12 mm, pulse width 50 milliseconds, fluence 25–35 J/cm^2^). It was noted that at 3 and 6 months of treatment, the mean hair removal efficiency was 74% and 79%, respectively [20].

### Nd:YAG Laser (1,064 nm)

The Q-switched 1,064-nm Nd:YAG laser with or without topical carbon suspension was one of the first laser systems used to remove hair. The poor absorption by melanin at this wavelength coupled with an epidermal cooling device makes the long-pulsed Nd:YAG laser a safe treatment option for patients with the darkest skin phototypes (III-VI) and, therefore, for darker Fitzpatrick skin types the long-pulsed Nd:YAG is preferred to the ruby laser [21,22]. Overall, clinical studies have demonstrated less hair reduction with the Nd:YAG laser compared with those results published with the ruby or alexandrite lasers [23]. A recent study by Rogachefsky et al evaluated the efficacy of a long-pulsed Nd:YAG laser system. A cryogen spray-cooled long-pulsed Nd:YAG laser was used to treat 22 subjects. Four adjacent sites were assigned to each subject and were treated with parameters of 50 J/cm^2^ with a 25-millisecond pulse duration, 60 J/cm^2^ with a 50-millisecond pulse duration, 80 J/cm^2^ with a 50-millisecond pulse duration, and control. Hair counts were obtained immediately and 1 week, 1 month, and 3 months after treatment, and multivariate regression analysis was used to determine the significance of hair reduction. At 3 months, the higher settings of 60 J/cm^2^ and 50 milliseconds and 80 J/cm^2^ and 50 milliseconds were statistically significant for reduced mean hair counts while the lowest setting at 50 J/cm^2^ and 25 milliseconds was not significant [24].

**Figure 3:**
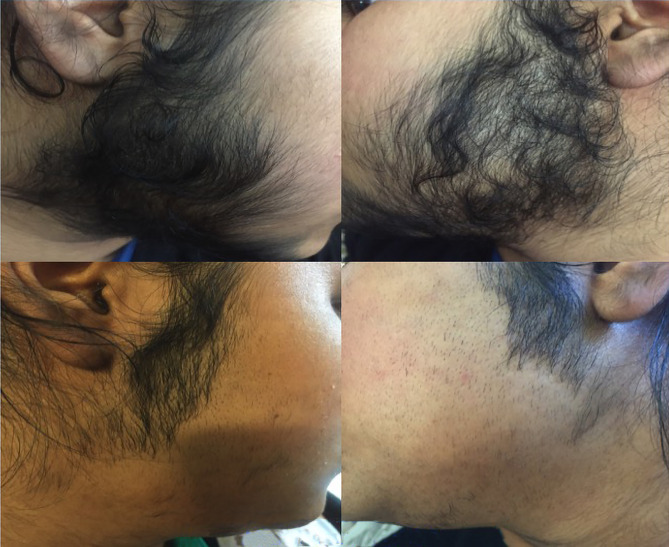
Hair reduction in a patient after 2 sessions of Nd:YAG laser hair removal.

**Figure 4:**
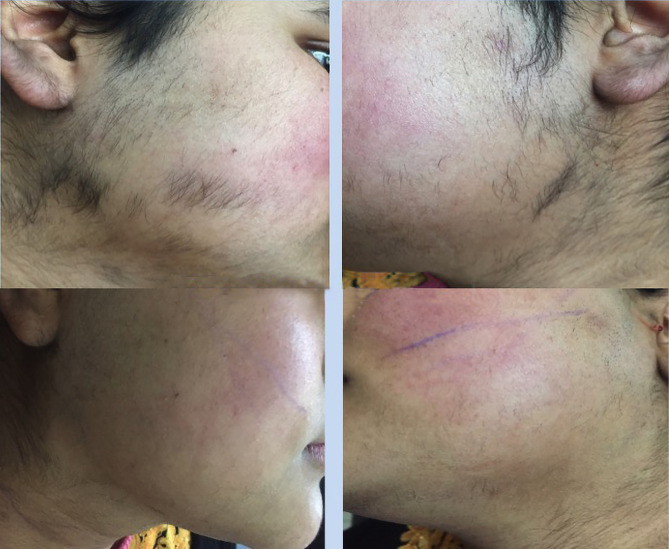
Significant hair reduction in the same patient after 4 and 6 sessions of Nd:YAG laser hair removal.

Figures 3 and 4 show significant hair reduction in a patient after 6 sessions of Nd:YAG laser hair reduction.

### Intense Pulsed Light (550–1,200 nm)

This system delivers broad-spectrum, noncoherent radiation with wavelengths of 550–1,200 nm. One of 4 filters (590 nm, 615 nm, 645 nm, or 695 nm) is used to eliminate shorter wavelengths. In general, filters with higher cutoff values are used with darker skin types [25,26]. Treatment with intense pulsed light may be useful for light-colored hair, although more treatment sessions are generally required. In one study, 60% hair reduction was reported 12 weeks after a single treatment with various cutoff filters (34–44 J/cm^2^, 2–5 pulses, 1.5–3.5 milliseconds, 20–50 milliseconds delays) [27,28].

### New Combination

Trio is a single multiplex diode laser hand piece that offers the synergistic benefits of the 3 most effective wavelengths for hair removal.

#### ALEX 755 nm Wavelength

The superficial penetration of 755 nm wavelength targets the bulge of the hair follicle and has thus been found to be more effective for superficially embedded hair in areas such as the eyebrows and upper lip.

#### SPEED 810 nm Wavelength

It is especially helpful because of its deep penetration capabilities that target the bulge and bulb of the hair follicle. It is also ideal for treating the arms, legs, beard, and cheeks by moderate tissue depth penetration.

#### YAG 1,064 nm Wavelength

As this wavelength offers the deepest penetration of hair follicle, it targets the bulb and the papilla and also treats the deeply embedded hair in areas like the scalp, axillae, and pubic areas.

In summary, the ruby laser (694 nm), alexandrite laser (755 nm), diode laser (800 nm), intense pulsed light source (550–1,200nm), and the Nd:YAG laser (1,064 nm), with or without the application of carbon suspension, work on the principle of selective photothermolysis with the melanin in the hair follicles as the chromophore. Multiple treatments are necessary to achieve satisfactory results regardless of the type of laser used. After repeated treatments, hair clearance of 30%–50% is generally reported 6 months after the last treatment. Patients with dark skin (Fitzpatrick skin types IV and V) can be treated effectively with morbidity comparable with those with lighter skin.

## Comparison of Different Laser and Light-Based Devices

The majority of studies have documented a superior efficacy of alexandrite and diode laser systems in hair removal in comparison with other lasers or light-based devices. In a study that compared the efficacy of 3 laser devices, a mean hair reduction of 59.5%, 70.3%, and 47.4% was reported after 3 sessions with diode, alexandrite, and Nd:YAG lasers, respectively [29,30]. In a randomized, split-face study, a mean reduction in hair count of 46% and 27% was reported after alexandrite and intense pulsed light systems, respectively [31].

In a comparative study on long-pulsed Nd:YAG laser and intense pulsed light system in skin types IV-VI, the former device was found to be more effective than the latter for hair removal, with fewer side effects. In darker skin types, a superior efficacy of Nd:YAG laser over intense pulsed light system has been demonstrated [14].

## For Light, White, and Gray Hair

Laser hair removal is based on targeting melanin. Absence of melanin or decreased melanin often results in failure of treatment. Goldberg et al have used combined light/bipolar radiofrequency devices along with photosensitizers with some success, suggesting that photosensitizers augment the effects of combined radiofrequency devices in nonpigmented hair reduction [32]. A recent alternative approach has been external application of melanin to the hair through the use of liposomal technology. Meladine, a topical melanin chromophore, has been studied in Europe with interesting results. Sand et al utilized melanin-encapsulated liposomes to improve performance of laser hair removal in nonpigmented hair [33].

## Future Technology Advances

Pneumatic skin flattening is a technique of reducing pain during the procedure. It works on gate theory of pain transmission wherein immediately prior to laser pulse it stimulates pressure receptors with the help of a vacuum chamber by generating a negative pressure and flattening the skin [34].

Other than laser, alternative technologies such as electro-optical synergy technology have been used. Electro- optical synergy technology combines electrical and optical lights. First the hair shaft is heated by laser/light energy, which then is thought to concentrate the bipolar radiofrequency energy to the surrounding hair follicle. This has an advantage of the use of less fluence and hence less epidermal damage and can also be effective in poorly pigmented hair [35].

### Total Reflection Amplification of Spontaneous Emission of Radiation

A Total Reflection Amplification of Spontaneous Emission of Radiation (TRASER) is not a laser; it is fundamentally different, and, in many ways, much simpler in design. It utilizes the energy from, for example, a flash lamp to induce the spontaneous emission of photons from a fluorescent dye in solution, or ions impregnated in a crystal structure. The light generated can be tuned from UVA to near infrared. Response of hair follicles to this mode of energy delivery has been studied wherein the follicular structures were targeted with dye cell switched to sulforhodamine 640 chloride, producing a narrow peak of 654 nm. Single 20-millisecond pulses with a 12-mm spot size and fluences from 14 to 20 J/cm^2^ were fired. Chest hair was treated with contact cooling.

Clinical and histological punch biopsy at 30 minutes after hair treatment observations reflected a clinical end point of perifollicular edema and transient erythema. Histologically, the damage was shown to be limited to the target structures. Clinical and histological acute phase changes associated with the use of TRASER have shown important observations; thus it can be used as a novel and effective method of hair removal [36].

### Vacuum-Assisted Laser Hair Removal

In vacuum-assisted laser hair removal, a vacuum gently draws the skin into the hand piece; skin is stretched thin and thereby the target is pulled closer to the energy source. Energy when applied to the target leads to spreading apart of the melanocytes and constriction of blood flow. Target is damaged and skin is released [37].

### Large Spot Size

Vectus (Palomar) is a hair removal system that uses a diode laser. It has a large spot size, which makes large areas such as back of legs easier to treat. The large spot size provides efficacious hair removal due to photon recycling and increased depth of penetration. In a study, 18-mm spot size was more effective than 12-mm spot size [37].

### Super Hair Removal

The original concept of progressive photothermolysis is included in the operation of the diode laser super hair removal (SHR) for photoepilation. During treatment, pulse repetition of the laser system SHR is 10 Hz. Depilation is performed through the continuous movement of the hand piece over the skin surface while delivering laser pulses, which prevents energy from concentrating on a particular point and thus generating burns due to overheating. Due to the relatively long-duration pulse of the laser SHR and its high repetition frequency, the laser energy penetration is secondarily increased and reaches deeper into the skin through a thermal propagation mechanism [38].

## Conclusions

The use of lasers/light-based technology in the treatment of unwanted hair has become common. The acceptance of photoepilation by both physicians and patients is a direct reflection of the high degree of efficacy, few side effects, and few complications. The benefits of this technology, however, have largely been limited to individuals with dark hair and relatively fair skin. The major challenge in the field of photoepilation continues to be the development of technology that not only permanently and significantly reduces the number of hairs but also provides permanent and complete hair removal for all skin and hair types and colors. With current technology, the average clearance rate is 20%–75% after 1–6 months of follow-up. Long-term studies with a follow-up of more than 1 year are needed to determine whether permanent hair removal can be accomplished. With the rapid pace of technological advancements and continued studies of hair biology, laser physics, skin optics, and cooling means, it is anticipated that permanent hair removal will be achieved in the near future.
